# Translational methods to detect asymmetries in temporal and spatial walking metrics in parkinsonian mouse models and human subjects with Parkinson’s disease

**DOI:** 10.1038/s41598-019-38623-6

**Published:** 2019-02-21

**Authors:** Lauren Broom, Audrey Worley, Fay Gao, Laura D. Hernandez, Christine E. Ashton, Ludy C. Shih, Veronique G. VanderHorst

**Affiliations:** Department of Neurology, Division of Movement Disorders, Beth Israel Deaconess Medical Center and Harvard Medical School, 3 Blackfan Circle, Boston, MA 02115 USA

## Abstract

Clinical signs in Parkinson’s disease (PD), including parkinsonian gait, are often asymmetric, but mechanisms underlying gait asymmetries in PD remain poorly understood. A translational toolkit, a set of standardized measures to capture gait asymmetries in relevant mouse models and patients, would greatly facilitate research efforts. We validated approaches to quantify asymmetries in placement and timing of limbs in mouse models of parkinsonism and human PD subjects at speeds that are relevant for human walking. In mice, we applied regression analysis to compare left and right gait metrics within a condition. To compare alternation ratios of left and right limbs before and after induction of parkinsonism, we used circular statistics. Both approaches revealed asymmetries in hind- and forelimb step length in a unilateral PD model, but not in bilateral or control models. In human subjects, a similar regression approach showed a step length asymmetry in the PD but not control group. Sub-analysis of cohorts with predominant postural instability-gait impairment and with predominant tremor revealed asymmetries for step length in both cohorts and for swing time only in the former cohort. This translational approach captures asymmetries of gait in mice and patients. Application revealed striking differences between models, and that spatial and temporal asymmetries may occur independently. This approach will be useful to investigate circuit mechanisms underlying the heterogeneity between models.

## Introduction

Gait problems in Parkinson’s disease (PD) may manifest in multiple ways, including shuffling, freezing of gait, festination, and asymmetries^1–4^. Gait asymmetries do not necessarily correlate with other asymmetries of motor signs in PD as assessed using the Unified Parkinson’s Disease Rating Scale (UPDRS)^5^. They affect a subset of patients^6^ and are heterogeneous, as they can be spatial or temporal in nature^4–9^. Gait asymmetries in PD may contribute to impairments like freezing of gait yet remain poorly understood^10^. Additionally, there has been considerable variation in measures that have been reported to be asymmetric in PD compared to control subjects, including step length^8,9,11^, swing time^4–8^, stance time^6^ and step time^6,8,11^, while others report no differences in step length^6^, or stance time^9^. These variable results may relate to differences in methodology or differences in subject populations. More standardized measures of gait asymmetries could potentially serve a role as part of a set of biomarkers for PD subtype or as a measure of disease progression or intervention.

The pathophysiology underlying gait asymmetries in PD remains unknown. As approaches to study underlying pathophysiology in living human subjects are limited^12^, translational models, including mice, offer a means to unravel circuit mechanisms underlying different types of gait asymmetries. This could include selecting appropriate sites for intervention and to evaluate rehabilitation, neuromodulation or gene therapy treatments to selectively correct abnormal circuit function. However, for such a line of translational research to be successful, a methodology is required that captures asymmetries of gait metrics both in subjects with PD and in mouse models within a relevant speed range, i.e. at walking speeds in human subjects, as these relate to patient outcomes, and at speeds that represent walking or trotting in mice, where hindlimb coordination is similar to walking in humans. An additional requirement is availability of experimental models that exhibit clinically relevant asymmetries of gait metrics.

As for methodology, there is no standardized approach to measure gait asymmetries in humans nor in mouse models of experimental parkinsonism. It is also unclear which mouse models of experimental parkinsonism are most suitable to produce gait asymmetries. For example, systemic injections of 1-methyl-4-phenyl-1,2,3,6-tetrahydropyridine (MPTP) did not cause asymmetry in swing duration or stride length^13,14^. Similarly, in a bilateral 6-hydroxydopamine (6-OHDA) model, striatal injections did not induce asymmetries in temporal hindlimb metrics, although these injections altered quadruped-specific temporal footfall patterns^15^. In mice, a unilateral version of the 6-OHDA model induced asymmetries in forelimb motor tasks^16^, but there are no reports on the effects on gait symmetry. In the rat, the unilateral 6-OHDA model has been shown to induce asymmetries in forelimb performance during motor tasks^17,18^ as well as asymmetries in spatial or temporal gait metrics^19–23^.

To address the need for standardization, in this study we present approaches to visualize and quantify asymmetries in temporal and spatial gait metrics in mouse models and human subjects. In mice, we first measure asymmetries within a condition by adapting a regression analysis based method that we previously validated to quantify changes in gait metrics within a speed range that is clinically relevant, i.e. at walking speed^24^. This method revealed that speed dependent gait signatures changed subtly in a unilateral 6-OHDA model and robustly in a systemic MPTP model. However, this prior approach was not designed to detect asymmetries in gait metrics as it pooled left and right datasets. Here we extend this approach by comparing left and right datasets of spatial and temporal gait metrics at walking speed in these two mouse models. Furthermore, to quantify asymmetries between conditions, i.e. baseline versus experimental parkinsonism, we applied circular statistics to datasets visualized in polar plots, as commonly performed in studies on rhythmic locomotor activity^25–27^. Finally, to determine whether similar methodologies also detect asymmetries in gait metrics of subjects with PD but not control subjects and whether such asymmetries resemble those detected in PD mouse models, we applied this approach to gait datasets of human subjects with PD and healthy age matched controls.

## Results

### Gait asymmetries at walking speed in parkinsonian mice

We used mouse models of parkinsonism that result in a unilateral (6-OHDA) or bilateral (MPTP) loss of striatal dopaminergic innervation, along with a control (untreated) group to test the validity of three types of analysis, expecting gait asymmetries in the unilateral but not bilateral or control groups.

In a first series of analyses, we tested whether right and left step length, stride length, swing time, and stance time as a function of stride velocity were similar using regression analysis in the baseline and the parkinsonian condition. In this analysis we fit the datasets of each of the gait metrics to the simplest fitting regression model in the speed range of 3–16 cm/s. We then used an F test to determine whether the regression curves representing the two datasets were shared.

#### Group 1: 6-OHDA

**Figure 1:**
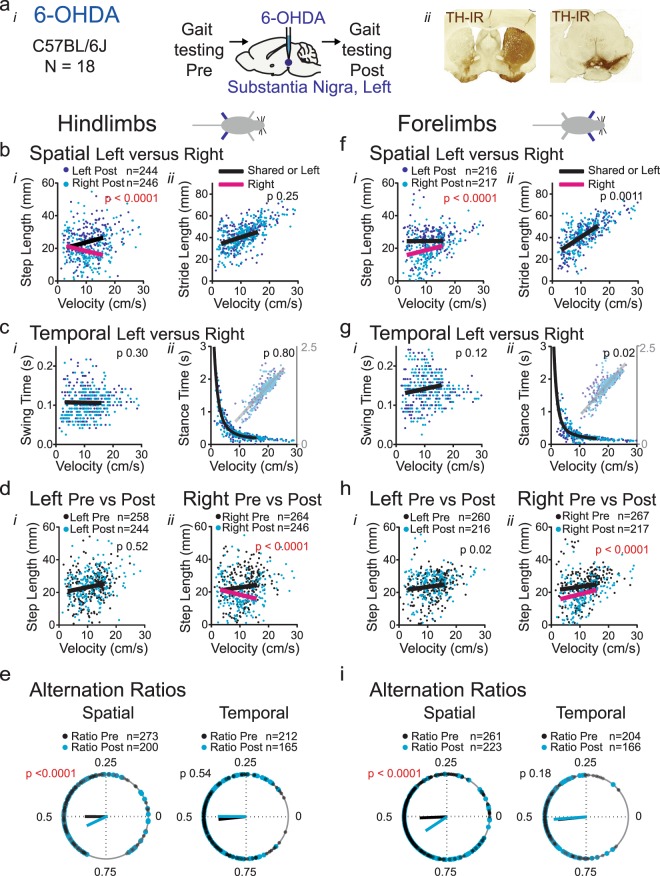
Spatial and temporal measures of gait symmetry in a unilateral 6-OHDA model a: Gait was tested before and after injection of 6-OHDA into the left substantia nigra (*i,ii right*) and cases included for analysis (n = 18) had >80% loss of tyrosine hydroxylase immunoreactivity (TH-IR) in the ipsi- compared to the contralateral striatum (*ii left*). (**b,e**) Hindlimb gait metrics. Spatial metrics step length (*bi*) and stride length (*bii*) and temporal metrics swing time (*ci*) and stance time (*cii*) of the left (dark blue dots) and right (cyan dots) hindlimbs as a function of velocity following induction of parkinsonism. (**d**) Spatial metrics step length of the left (*i*) or right (*ii*) hindlimb as a function of velocity before (black dots) and after (blue dots) induction of parkinsonism. In (**b**–**d**) a combination of black and magenta lines represent a significant difference in datasets. A single black line represents the curve that is shared among datasets when they are not significantly different (for linear models F-test captures both slope and Y intercept); p value set at 0.001). Analyses included only datasets from the same mice (within group comparisons between limbs or conditions). (**e**) Spatial and temporal alternation ratios of the hindlimbs were plotted on the circular axis of polar plots, with resultant vectors indicating mean value (angle of line) and strength of mean (length of line). Black dots represent ratios before and cyan dots ratios after induction of parkinsonism. The Watson-Williams test was used to determine differences in group means (p value set at 0.05; Supplemental Table 3S). (**f–i**) Forelimb gait metrics of the same mice as in panels b-e, with legends for (**f–i**) corresponding to those of (**b–e**). Analyses included only datasets from the same mice (within group comparisons between limbs or conditions).

As expected, in the baseline condition, there were no differences between left and right side in hindlimb step or stride length, and in stance or swing time at walking speed (Supplemental Table 1S) (Fig. 1a). Following 6-OHDA treatment, we measured an asymmetry in step length with the shorter step length contralateral to the 6-OHDA injection (Fig. 1b, magenta; Supplemental Table 1S). There were no differences between left and right hindlimb stride length, nor in swing and stance time (Fig. 1b,c; Supplemental Table 1S). Using the same regression model or a paired t test to analyze these same datasets, but averaged for each mouse, a significant difference in step length was found (Supplemental Fig. 1S, Supplemental Tables 2S and 3S). The significant difference found for stance duration in the baseline condition when using the t-test (t = 2.38, df = 17; p 0.03; Supplemental Table 3S) illustrates that this test is not appropriate for stance data, which behaves logarithmically as a function of speed.

To confirm these findings we then compared step length between baseline and post-lesion conditions within the same limb. We found no differences for the left hindlimb data but verified the shorter step length of the right hindlimb, the side contralateral to the lesion, in the post-lesion condition (Fig. 1d, magenta; Supplemental Table 4S). The forelimb datasets behaved similarly to hindlimb datasets both in the baseline and post-lesion conditions (Fig. 1f–h; Supplemental Table 1S and 4S). This indicates that unilateral loss of nigral dopaminergic innervation results in a step length asymmetry affecting hind- and forelimbs, with shortening contralateral to the lesion.

#### Group 2: MPTP

**Figure 2:**
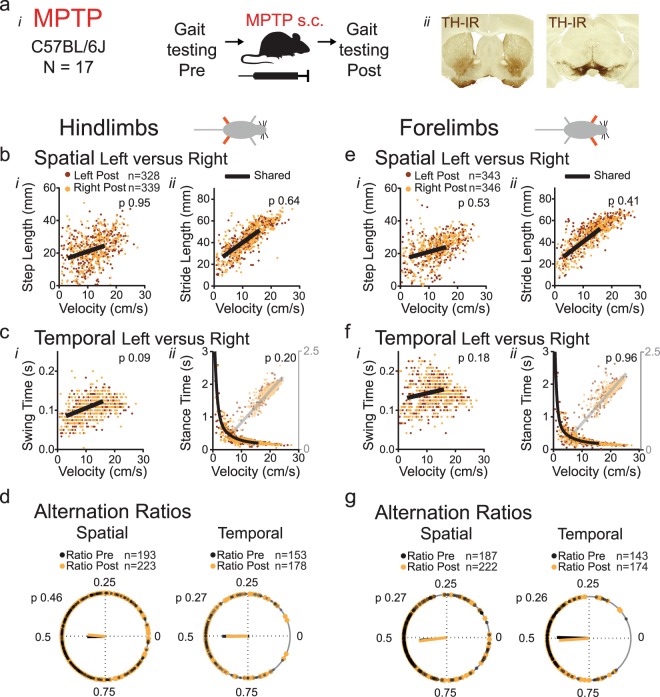
Spatial and temporal measures of gait symmetry in a systemic MPTP model (**a**) Gait was tested before and after systemic injection of MPTP (*i*) and cases included for analysis (n = 17) had >65% loss of tyrosine hydroxylase immunoreactivity (TH-IR) in the striatum (*ii*) compared to control (Fig. 4a). (**b–d**) Hindlimb gait metrics. Spatial metrics step length (*bi*) and stride length (*bii*) and temporal metrics swing time (*ci*) and stance time (*cii*) of the left (red dots) and right (orange dots) hindlimbs as a function of velocity following induction of parkinsonism. Single black lines indicate that datasets share the same curve (F-test, p value set at 0.001; Supplemental Tables 1S and 2S). (**d**) Spatial and temporal alternation ratios of the hindlimbs were plotted on the circular axis of polar plots, with resultant vectors indicating mean value (angle of line) and strength of mean (length of line). Black dots represent ratios before and orange dots ratios after induction of parkinsonism. The Watson-Williams test was used to determine differences in group means (p value set at 0.05; Supplemental Table 3S). (**e–g**) Forelimb gait metrics of the same mice as in panels b-d, with legends for (**e–g**) corresponding to those of (**b–d**).

We found no asymmetries in hindlimb gait metrics at walking speed prior to MPTP treatment (Supplemental Table 1S) (Fig. 2a). Following systemic MPTP treatment, in contrast to the 6-OHDA model, step length did not differ between right and left sides (Fig. 2b; Supplemental Table 1S). There was also no difference between left and right hindlimb stride length, swing or stance time (Fig. 2b,c; Supplemental Table 1S). Similarly, we detected no asymmetries in gait metrics of the forelimbs (Fig. 2e,f). These data indicate that systemic MPTP does not result in asymmetries in spatial or temporal gait metrics between left and right hind- or forelimbs.

#### Group 3: Control

**Figure 3:**
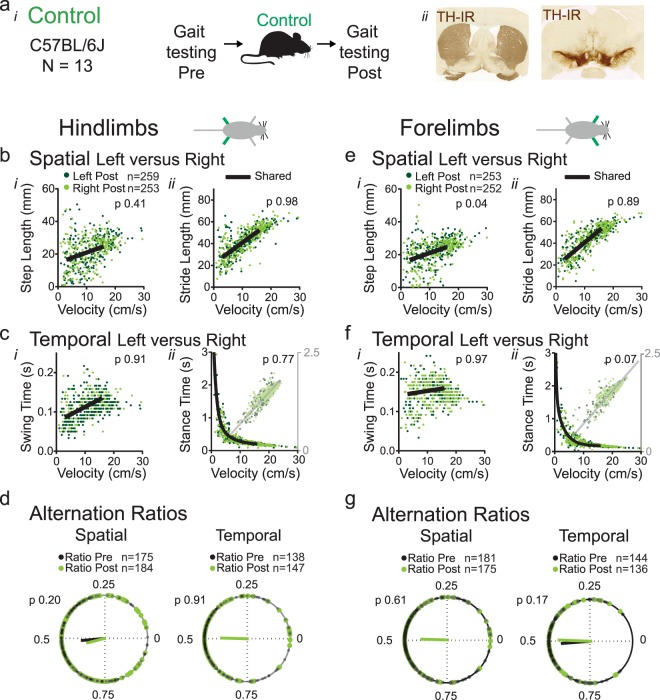
Spatial and temporal measures of gait symmetry in a control model. (**a**) Gait was tested twice without induction of parkinsonism (*i*) and all 13 cases were included for analysis (n = 13). These also served to obtain baseline densities of tyrosine hydroxylase immunoreactivity (TH-IR) in the striatum (*ii*) for the MPTP group (Fig. 3). (**b**–**d**) Hindlimb gait metrics. Spatial metrics step length (*bi*) and stride length (*bii*) and temporal metrics swing time (*ci*) and stance time (*cii*) of the left (black dots) and right (blue dots) hindlimbs as a function of velocity at two different time points. Single black lines indicate that datasets share the same curve (F-test, p value set at 0.001; Supplemental Tables 1S and 2S). (**d**) Spatial and temporal alternation ratios of the hindlimbs were plotted on the circular axis of polar plots, with resultant vectors indicating mean value (angle of line) and strength of mean (length of line). Black dots represent ratios of test one and green dots ratios of control test two. The Watson-Williams test was used to determine differences in group means (p value set at 0.05; Supplemental Table 3S). (**e–g**) Forelimb gait metrics of the same mice as in panels b-d, with legends for (**e–g**) corresponding to those of (**b–d**).

We detected no differences in hind- or forelimb step length, stride length, swing or stride time between right and left sides in non-treated mice in any of the 2 testing sessions (Fig. 3b–f; Supplemental Table 1S) (Fig. 3). This strongly suggests that the methodology specifically detects changes due to underlying dysfunction of motor circuitries.

### Comparing gait asymmetries between baseline and parkinsonian conditions

**Figure 4:**
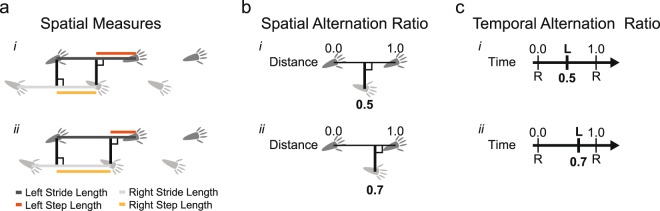
Schematic depiction of measures used to determine symmetry of foot placement and timing during walking. (**a**) Step length is calculated as the distance at which one limb is placed in front of the opposing limb. This is distinct from stride length, which refers to the distance between subsequent foot placements of the same limb. Symmetric foot placement (*i*) is characterized by equal step length on left and right sides, whereas asymmetric placement (*ii*) results in unequal step length on the left compared to the right side. (**b**) Spatial alternation ratio represents foot placement relative to the length of a stride on the opposite homologous limb. Symmetric gait is characterized by a ratio of 0.5 (*i*) and asymmetric gait by a ratio that deviates from this (*ii*). (**c**) Temporal alternation ratio represents timing of footfall relative to timing of prior and subsequent footfalls on the opposite homologous limb. Symmetric gait is characterized by a temporal alternation ratio of 0.5 (*i*) and asymmetric gait by a ratio that deviates from this (*ii*).

The above method enables comparison of left and right gait metrics within a condition or of gait metrics of a single limb between conditions in a speed-dependent manner, but not of gait metrics from paired limbs between conditions. To address this limitation, we implemented spatial and temporal alternation ratios that capture the spatial or temporal offset between paired limbs (Fig. 4b,c). We then visualized data-points of baseline and post-lesion condition in polar plots, and used circular statistics to measure differences between conditions.

#### Group 1: 6-OHDA

Spatial alternation ratios of hindlimbs differed significantly between baseline and post-lesion condition (Fig. 1e,i; Supplemental Table 5S), illustrated in the polar plots as difference in mean direction. The same was true for forelimb spatial alternation ratios. This confirms the results of the speed dependent analyses reported above, i.e. unilateral loss of nigral dopaminergic innervation results in an asymmetry in placement of pairs of hind- and forelimbs. As the data from the first analysis showed that the curves for left and right step length in the 6-OHDA condition diverged especially at the higher speeds within the 3–16 cm/s range, we then performed an additional analysis in which we divided the data in two speed bins (3–10 or 10–16 cm/sec). This analysis confirmed that spatial alternation was significantly different at 10–16 cm/sec, but not at 3–10 cm/s (Supplemental Fig. 4S, Supplemental Table 6S). We detected no difference in temporal alternation ratios of hind- or forelimbs between baseline and post-lesion conditions in the 3–16 cm/s range (Fig. 1e,i; Supplemental Table 5S) or the 3–10 cm/s or 10–16 cm/s ranges (Supplemental Table 6S).

#### Group 2: MPTP

In line with the results of the regression analysis, we detected no difference in spatial or temporal alternation ratios of hind- or forelimbs (Fig. 2d,g; Supplemental Table 5S; add 2 speed bins) between baseline and post-lesion conditions.

#### Group 3: Control

Spatial and temporal alternation ratios of pairs of hindlimbs or forelimbs did not differ between two control assessments (Fig. 3d,g; Supplemental Table 5S; add 2 speed bins). The absence of changes in control mice supports the validity of the methodology.

### Gait asymmetries in subjects with PD

**Figure 5:**
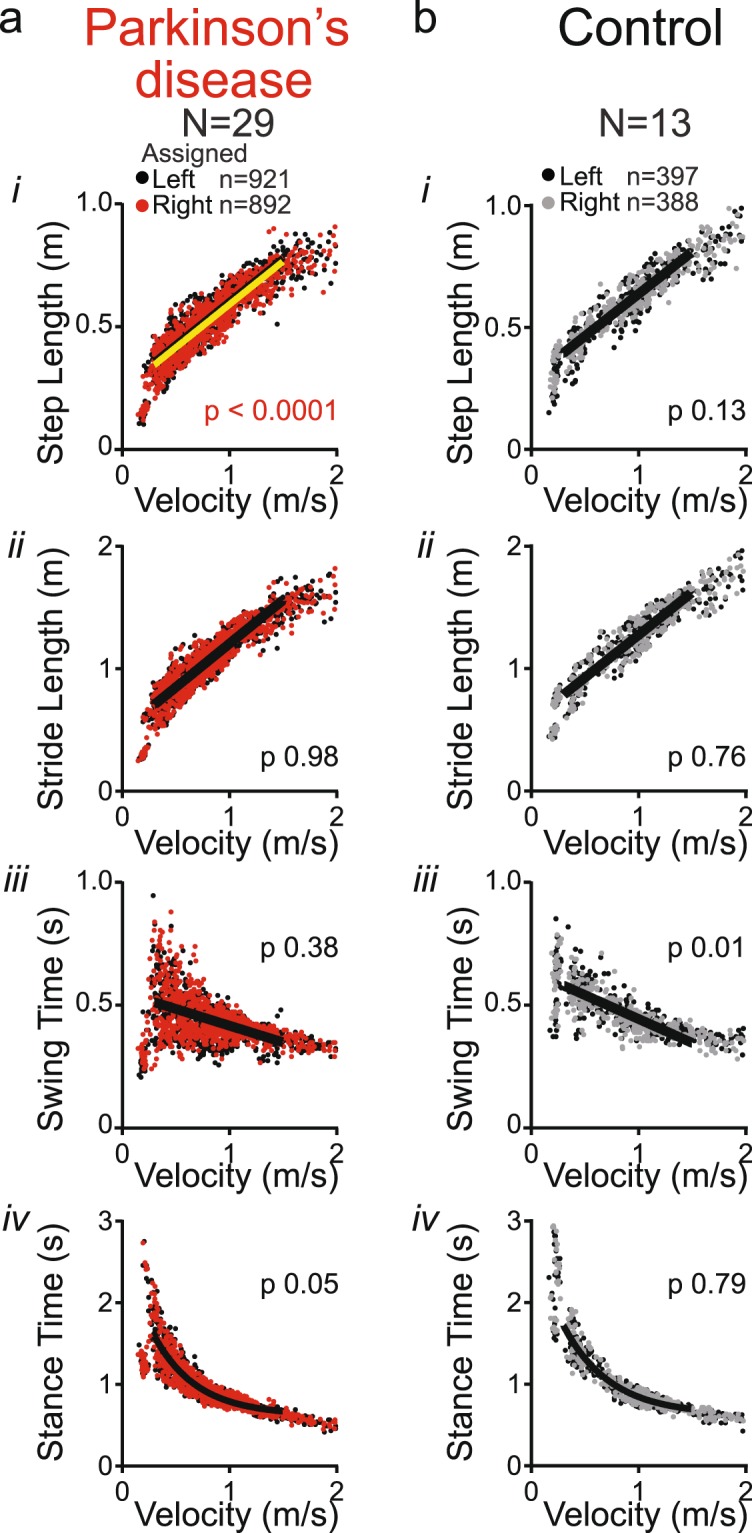
Spatial and temporal measures of gait symmetry in PD and control subjects Spatial and temporal metrics of the left and right legs from ground-walking trials in subjects with PD (**a**) and control subjects (**b**): (*i*) Step length, (*ii*) stride length, (*iii*) swing time and (*iv*) stance time as a function of stride velocity. Black dots represent metrics from the assigned left leg and red or gray dots of the assigned right leg. Single black lines indicate that left and right datasets share the same curve. A combination of black and yellow lines indicate that datasets of left (black) and right (yellow) leg differ significantly (F-test, p value set at 0.001; Supplemental Table 5S).

In the second part of this study, we set out to analyze datasets from male subjects with PD (n = 29) and age matched controls (n = 13), using an approach similar to analysis 1 for the mouse cohorts. However, in contrast to experimentally controlled mouse models, asymmetries in PD subjects are expected to randomly affect left or right side, making a comparison of datasets representing true left and right sides invalid. For the purpose of this study, we circumvented this problem by assigning the shorter step length of PD subjects with a visually obvious asymmetry (9 of 29) to the right side. For PD subjects without an asymmetry (20 of 29) and for controls (all 13), all of whom were classified as symmetric, we organized gait metrics per true left and right sides. In the entire PD cohort of 29 subjects, similar to the 6-OHDA mouse model, we found a significant difference between left and right step length, but we detected no stride length, swing time and stance time asymmetries (Fig. 5a; Supplemental Table 7S). We found no significant differences between left and right datasets in the control cohort (Fig. 5b; Supplemental Table 7S). PD and control groups did not differ in age, weight, height or MoCA score (Table 1).

**Table 1:** Summary of clinical data.

Number of subjects	PD		Control		Welch’s T test	p
	29		13			
	Mean	SD	Mean	SD		
**Analysis 1: Parkinson’s Disease (PD) versus Healthy control**						
Age (years)	65.8	5.9	62.2	8.4	t = 1.37 (17)	0.19
Height (inches)	69.4	2.1	69.9	2.4	t = 0.64 (21)	0.53
Weight (kg)	83.7	12.6	88.4	7.3	t = 1.46 (34)	0.15
MoCA	26.2	2.8	27.6	1.9	t = 1.89 (34)	0.07
Disease duration (years)	8.1	4.4	—	—	—	—
Motor MDS-UPDRS III	30.4	8.2	—	—	—	—
L-DOPA equivalent dose (mg)	661.0	529.0	—	—	—	—
Hoehn & Yahr	2.2	0.5	—	—	—	—
Average Speed (m/sec)	0.91	0.20	0.96	0.17	t = 0.90 (28)	0.37
**Number of subjects**	**PIGD**		**TD**		**Welch’s T test**	**p**
	**12**		**15**			
	**Mean**	**SD**	**Mean**	**SD**		
**Analysis 2: PD: Postural Instability and Gait Disorder (PIGD) versus Tremor Dominant (TD)**						
Age (years)	65.8	5.1	65.0	6.6	t = 0.33 (25)	0.74
Height (inches)	69.2	1.9	69.5	2.3	t = 0.42 (24)	0.67
Weight (kg)	81.7	8.3	84.7	15.8	t = 0.63 (22)	0.54
MoCA	25.1	3.2	26.9	2.2	t = 1.65 (19)	0.12
Disease duration (years)	9.7	5.1	7.7	3.4	t = 1.18 (18)	0.25
Motor MDS-UPDRS III	30.8	8.4	30.1	8.9	t = 0.21 (24)	0.84
L-DOPA equivalent dose (mg)	999.0	549.0	479.0	358.0	t = 2.83 (18)	0.01
Hoehn & Yahr	2.5	0.4	2.0	0.4	t = 3.04 (24)	0.006
Average Speed (m/sec)	0.88	0.14	0.95	0.24	t = 0.87 (23)	0.39
**Number of subjects**	**Asymmetry**		**No Asymmetry**		**Welch’s T test**	**p**
	**9**		**20**			
	**Mean**	**SD**	**Mean**	**SD**		
**Analysis 3: PD: Clinical Asymmetry versus No Clinical Asymmetry**						
Age (years)	67.6	3.9	65.0	6.6	t = 1.3 (25)	0.21
Height (inches)	68.8	1.9	69.7	2.1	t = 1.2 (17)	0.25
Weight (kg)	87.0	11.8	82.2	13.0	t = 0.99 (17)	0.34
MoCA	26.4	2.9	26.2	2.8	t = 0.25 (15)	0.80
Disease duration (years)	8.1	4.6	8.1	4.5	t = 0.006 (15)	1.00
Motor MDS-UPDRS III	33.6	5.4	29.1	9.0	t = 1.67 (24)	0.11
L-DOPA equivalent dose (mg)	502.0	575.0	733.0	507.0	t = 1.03 (14)	0.32
Hoehn & Yahr	2.3	0.5	2.1	0.5	t = 1.25 (17)	0.23
Average Speed (m/sec)	0.95	0.23	0.89	0.19	t = 0.67 (13)	0.52

Demographic and clinical measures of subjects with Parkinson’s disease (PD) and healthy controls. Group analyses were conducted between healthy control and all PD subjects (Analysis 1), PD subjects with predominant postural instability and gait disorder and predominant tremor (Analysis 2), and PD subjects with and without a clinically obvious gait asymmetry. Group averages were compared using a 2-tailed Welch’s T test (significance level set p < 0.05).

**Figure 6:**
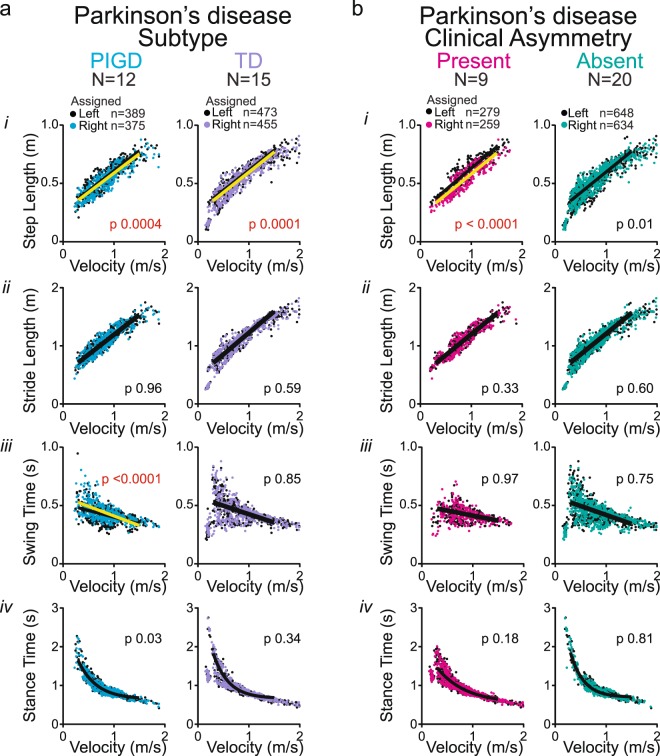
Spatial and temporal measures of gait symmetry in subgroups of PD subjects The PD group was further categorized into (**a**) Postural instability and gait dominant (PIGD) and tremor dominant (TD) subtype, or (**b**) with or without a clinically obvious gait asymmetry. (*i*) Step length, (*ii*) stride length, (*iii*) swing time and (*iv*) stance time as a function of stride velocity. Black dots represent metrics from the assigned left leg and colored dots of the assigned right leg. Single black lines indicate that left and right datasets share the same curve. A combination of black and yellow lines indicate that datasets of left (black) and right (yellow) leg differ significantly (F-test, p value set at 0.001; Supplemental Table 5S).

Next, we divided the PD cohort into postural instability and gait dominant (PIGD) and tremor dominant (TD) subgroups based on subjects’ scores on the MDS-UPDRS^28^ to assess whether the asymmetry in step length is a feature of PIGD or TD subtype. Fifteen subjects fell in the TD and 12 in the PIGD group. Two subjects were left out of this analysis, as their score placed them into an ‘indeterminate’ group. We then assessed whether the left and right datasets differed within each of the groups (Supplemental Table 7S). In both subgroups, we found the step length to be asymmetric (Fig. 6a*i*), suggesting that step length asymmetry is not a feature specific for PIGD or TD subtype. However, the PIGD group, but not TD group, showed a significant asymmetry for swing time (Fig. 6a*iii*). We found no stride length or stance time asymmetries in these groups (Fig. 6a*ii,iv*). The PIGD and TD groups differed significantly for L-DOPA equivalent dose and, as expected given the criteria for PIGD and TD subgroups, Hoehn & Yahr stage but not for age, weight, height, MoCA score, disease duration or MDS-UPDRS III score (Table 1).

In a final analysis, we divided the PD cohort into two groups solely based on clinical video assessment for asymmetry. In the group with a clinically obvious asymmetry in gait, step length was significantly asymmetric, but not swing time, stance time or stride length (Fig. 6b, Supplemental Table 7S). There were no differences between asymmetric and symmetric groups for age, weight, height, MoCA score, L-DOPA dose, MDS-UPDRS III, or Hoehn & Yahr stage (Table 1). The 9 clinically asymmetric subjects were evenly distributed among the PIGD (n = 4 subjects) and TD (n = 5) subgroups. In light of the prior analysis, this indicates that clinical video assessment in PD subjects preferentially captures spatial asymmetries and not temporal (i.e. swing time) asymmetries.

Swing time asymmetries and step length asymmetries did not correlate with asymmetries in bradykinesia or rigidity (Table 8S).

## Discussion

In this study, we present an approach to visualize and quantify left-right asymmetries in temporal and spatial gait metrics at walking speed that are relevant for subjects with PD and mouse models of experimental parkinsonism. Visualization of gait metrics as a function of speed followed by regression analyses enables comparisons of datasets from the left and right legs within an experimental condition or at a given time point. This approach can also be applied to comparisons of metrics of one leg over time, even if the range of gait speed changes over time, as is often the case in disease models. For questions related to an intervention induced effect on left-right symmetry, spatial and temporal alternation ratio scores and circular statistics are appropriate to detect changes in symmetry across conditions. Application of these methodologies to datasets from human subjects with PD or from various PD mouse models revealed asymmetries in spatial (step length) or temporal (swing time) gait metrics. Furthermore, such spatial and temporal asymmetries may occur independently and vary among mouse models of experimental parkinsonism and among clinically different human PD subtypes. Below we will discuss the technical, translational, and clinical implications.

### Different models of experimental parkinsonism produce different types of gait abnormalities

Comparing gait data obtained in mice and humans is important for the development of translational lines of research, but can be challenging given interspecies variation in size, speed range, differences in sensorimotor feedback and bipedal versus quadrupedal gait patterns^4,15,24,29–32^. Rather than developing a model that estimates the contributions of these variables, congruent visualization and interpretation of data from quadrupeds and humans becomes possible with use of speed-dependent spatial and temporal gait metrics such as stride and step length, swing and stance time, and cadence^32–37^. We recently developed and validated a method that makes use of these features to establish regression analysis based gait signatures at a speed range that has most clinical relevance, and to quantify differences in gait signatures between experimental conditions within mouse cohorts. By modifying this methodology to compare left and right datasets within a condition, we detected asymmetries in step length in a unilateral 6-OHDA model of experimental PD, concurring with reports from a similar model in the rat^21,22^. These robust asymmetries in step length following unilateral injections of 6-OHDA into the substantia nigra stand out from relatively modest changes of other gait metrics between baseline and experimental PD conditions in this model^24^.

In contrast to the unilateral 6-OHDA model, systemic MPTP exposure, which leads to bilateral neuronal loss, does not result in detectable asymmetries with the methodology presented. This supports results from other studies in the MPTP mouse model^13,14^ and is not surprising due to the bilateral nature of this model. Although we do not observe asymmetries in gait metrics in the MPTP model, this does not mean that gait is not affected in this model. Indeed, systemic MPTP significantly reduces stride length and swing time compared to baseline^24^ and is the more robust model for these specific deficits compared to the 6-OHDA model. However, as the systemic MPTP model does not produce an asymmetry in these metrics, it is not suitable when the goal of a study is to measure progression of asymmetry or the effects of an intervention on asymmetry. In other words, both the 6-OHDA and MPTP models can offer tremendous utility for studying interventions or therapies, but their utility depends on the specific aspect of dysfunction being studied.

When datasets are available of left and right sides at two different time points or conditions, alternation ratio scores and circular statistics allow for detection of changes in symmetry across conditions, as shown in the 6-OHDA dataset. This methodology is commonly used to measure temporal coupling between varied sets of limbs in locomotor research^26,27,38^. Polar plots have also been applied to treadmill gait data in a bilateral 6-OHDA mouse model and revealed an asymmetry of temporal coupling of diagonal pairs of limbs only^15^. Other than the forelimb stepping test in rats, the concept of asymmetries in spatial hind- and forelimb gait parameters is uncommon in research involving animal models. However, the phenomenon of visible spatial asymmetries, with one leg “leading”, is commonly observed by clinicians who see patients with Parkinson’s disease. This therefore represents an important entity from a translational perspective.

### Forelimb step length asymmetry during walking as a measure of limb preference

Data from forelimbs not only fit the model that we developed for hindlimbs but, similar to the hindlimb data, we found a step length asymmetry in forelimb datasets following 6-OHDA exposure. This finding has potential implications for testing paradigms in parkinsonian mouse models. Tests for asymmetries in limb use have been an integral part of PD rat and primate models, but these have been challenging to apply to mouse models. In rat models, a forelimb stepping test is commonly used to measure the length of adjusting steps while a restrained rat is moved by the experimenter across a flat surface. Though this test does not measure free-walking, it is performed at a slow speed (18 cm/s)^39^. Impaired rats take shorter steps on the side contralateral to the deficit^18,39,40^, which is similar to the findings in the unilateral 6-OHDA mouse model in the present study. Analysis of forelimb step length metrics during free walking may therefore provide a sensitive alternative measure to quantify asymmetries in forelimb use in PD mouse models.

### How do asymmetries in gait metrics translate between mouse PD models and PD subtypes in patients with PD?

Though a toolkit to measure gait deficits in parkinsonian mouse models is useful for basic and preclinical research, the measures that comprise it become even more meaningful when they have translational value. The results of this study show that it is feasible to analyze speed-dependent spatial and temporal gait parameters in human subjects in a similar way as in mouse datasets. This method does not formally compare mice and human data, but enable congruent visualization of data from both species to detect similarities and is important from a translational standpoint. As a group, PD subjects show a step length asymmetry similar to the 6-OHDA mouse model.

This step length asymmetry only involved a subset of PD subjects, similar to prior studies^12,41^. In the present study, these subjects were evenly distributed among TD and PIGD subtypes, indicating that this subtyping may not capture gait problems related to step length asymmetries. However, the same subtype analysis showed a swing time asymmetry in PIGD group only. This observation of an asymmetry in swing time in PIGD subjects agrees with results from a prior report in which a different methodology was used^6^.

Based on the comparison of demographic and global disease related datasets of subjects with and without step length asymmetry (Table 1), there were no indications of which major features may be associated with an asymmetric step length. With the limitation that our datasets were small, we also found no correlation between step length or swing time asymmetry and asymmetries in rigidity or bradykinesia as captured in the MDS-UPDRS. These observations are in line with findings of prior studies reporting that swing time asymmetry does not or not strongly correlate with UPDRS asymmetry^5,42^, but differ from a study in which step length asymmetry was shown to correlate with severity of parkinsonism^43^.

These combined findings implicate that gait asymmetries in PD are heterogeneous and can present independently with temporal or spatial features. This in turn suggests that different pathophysiological mechanisms underlie these features. The spatial features may well be due to asymmetric dopaminergic deficits, at least based upon similarities with the unilateral 6-OHDA mouse model. The asymmetry in swing time as seen in a subset of PD subjects is not reproduced by the systemic MPTP or unilateral 6-OHDA models. It was not surprising to observe that these classical models do not capture all features that can manifest in PD gait, given the widespread pathology in PD beyond the nigrostriatal system^44^. Further studies will be necessary to reveal which circuit dysfunction underlies this swing time asymmetry and whether other age-related conditions^45^ or other features of PD such as dyskinesia or dystonia may account for this phenomenon.

### Strengths and weaknesses of the study

This study has several strengths. Firstly, the methodology presented captures spatial as well as temporal asymmetries in gait metrics obtained from the same datasets. Secondly, the methods can be applied to mouse and human gait measures, as long as left and right datasets represent matched datasets within a cohort. This facilitates translational efforts and helps to select the most appropriate mouse model to represent features of gait dysfunction in Parkinson’s disease. This also points to feasibility of constructing a “translational toolkit” that allows for mapping of signs from relevant animal models to human subjects and back, and measuring changes in these signs whether due to the disease itself or due to interventions aimed to correct deficits. Thirdly, the methodology was validated in both control and disease conditions and can be extended to other diseases or disease models. Limitations of the study include that we only studied a subset of PD mouse models, and other models may reveal different patterns of gait asymmetry. In addition, the methodology has only been validated for the speed range of 3–16 cm/s, and adjustments would be required to extend the analyses to higher speed ranges. Finally, in the mouse studies, we only assessed hindlimb or forelimb coupling, as this is relevant for translational purposes. We did not assess coupling between other combinations of limbs as is common in locomotor studies, as our aim was to develop a toolkit of measures that are meaningful from a translational standpoint. It should also be noted that the presented methodology is not designed to model mouse or human gait in general^24^. Between species and among groups of mice with distinct genetic backgrounds, gait curves are similar, but not the same, which may be due to differences in length, sex and weight^24^, kinematics, sensorimotor delay and other variations in organization of central neural control^31,46–49^.

The human portion of the study has additional limitations. We manually assigned the presence and side of asymmetry to all subjects, prior to analyses, in order to organize the side with shorter metrics to the same side for all subjects. This may have introduced bias despite our efforts to blind raters in this clinical assessment. Future studies will be aimed to develop unbiased statistical approaches to detect abnormalities in gait metrics. Furthermore, the human datasets represented a relatively small number of exclusively male subjects. Though this limits the extent to which the results of the analyses can be generalized, it does not undermine the methodology itself.

### Impact

Analysis of temporal and spatial gait metrics provides insights into gait abnormalities in both human disease states and animal disease models. Equipment to obtain these metrics has become widely available and permits the production of vast datasets including many different gait measures. However, it has remained a challenge to determine which parameters are relevant for a particular question or disease condition, and how metrics translate between human disease and animal disease models. The resulting lack of standardized approaches that facilitate translational research adds to high failure rates of clinical trials^50^. The results of this study show that it is feasible to build a suite of clinically relevant translational gait assessment tools that are applicable to both mouse and human models. These translational methodologies can benefit future studies that focus on understanding circuit mechanisms underlying gait disorders or that evaluate the efficacy of potential treatments in animal models or in humans. Furthermore, at the individual level, the methodology may provide a precise metric to quantify progression over time, as the interpretation is not affected by a decline in speed over time. Finally, as no mouse model perfectly replicates Parkinson’s disease, selecting a disease model appropriate to the specific aspect being studied is crucial. With the examples of the MPTP model representing global and symmetric shifts in gait signatures, and the 6-OHDA model representing spatial asymmetries, this study illustrates the importance and feasibility of model selection based on the specific symptom targeted for study.

## Methods

### Mouse study

#### Animals

Housing, handling, behavioral tests, and surgery and post-operative monitoring were performed according to the Guide for the Care and Use of Laboratory Animals at the Animal Research Facility at the Center for Life Sciences, Beth Israel Deaconess Medical Center (BIDMC). Study protocols were reviewed and approved by the IACUC at BIDMC.

We used 64 male C57BL/6 J mice, which were shipped from Jackson Labs at 8–10 weeks of age and were in the mature age range when tested. They were group-housed (except when isolated due to fighting), with free access to food and water under a 12 hour light/dark cycle. Since we have not previously performed the analyses used throughout this paper, we could not perform power analyses prior to the study to determine adequate group size. Therefore we used group sizes similar to those of a preceding study in which we used pooled left/right hindlimb data^24^. In the current study we analyzed relevant subsets of data from this preceding study. We focused on male mice given sex differences in gait metrics^24^ and in sensitivity to toxins for the induction of parkinsonism^51^, which reach beyond the scope of the current methodological study.

Group 1: *6-OHDA:* For the induction of unilateral parkinsonism with 6-hydroxydopamine (6-OHDA) we used 32 mice. Under aseptic conditions, we injected 6-OHDA (Sigma; 3 µg/µl) into the substantia nigra with a glass micropipette using a stereotactic approach under isoflurane anesthesia as previously described^24^. Meloxicam analgesia was administered at the start of the surgery and 24 hours later. We tested mice 1–2 weeks prior to and 3–5 weeks following 6-OHDA injections. Fourteen mice in this cohort were excluded based upon insufficient nigral cell loss as determined through post-hoc histological analysis (see histological processing and analysis below).

Group 2: MPTP: For the induction of bilateral parkinsonism with systemic 1-methyl-4-phenyl-1,2,3,6-tetrahydropyridine (MPTP) we used 19 mice. We injected MPTP (Sigma, 25 mg/kg) subcutaneously for 5 consecutive days as described previously^24^. We tested mice both 1–2 weeks prior to and 4 weeks after the last MPTP injection. Two mice in this cohort were excluded based upon insufficient nigral cell loss as determined through histological analysis (see histological processing and analysis below).

Group 3: Thirteen mice served as a control group. These mice did not receive 6-OHDA or MPTP injections and were tested at 2 separate time points.

#### Gait testing

Testing was performed during the first 4 hours of the light-ON phase, on non-consecutive days only. After habituation, animals were tested on a custom-made runway as previously described^24^. We obtained videos (120 frames per second with 1/1000 sec shutter time) of mice that walked across the middle portion of the runway. For each condition, 4–8 walking trials were necessary to capture sufficient data. One experimenter completed testing of all conditions in the MPTP and control cohorts. The trials for the 6-OHDA group were collected by a different tester.

#### Video gait analysis

Raters blinded to the experimental condition^24^ scored video frames to extract temporal and spatial gait data. Blinding was achieved by providing the raters with only the mouse number along with an automatically generated video file name. Videos were spatially calibrated and analyzed using custom MATLAB software as previously described^24^. A rater marked the location of each paw placement and the associated first frames of the stance and swing phases. We then calculated stride velocity, stride length, swing time, stance time for each stride of each limb. This was done for 4 trials per mouse. As described previously, the exact speed range used for analysis was further decided by performing an F-test in a large number of different mouse cohorts to determine which models fit the data at the full velocity range and then data for limited segments of stride velocity (see below^24^).

#### Statistical analyses

We used 3 types of analyses to examine asymmetries in gait metrics. All comparisons were done within the same group (e.g. data from each mouse had associated baseline and experimental conditions and/or data from left and right limbs). Analyses were done for 3 groups:

Analysis 1: Quantifying asymmetries within a condition: To detect asymmetries in gait metrics within a condition while taking into account the speed dependent nature of each of the gait metrics, we modified an approach that we previously validated to compare gait metrics of the same mice between conditions^24^. We used this approach to compare data from one limb across conditions or data from 2 limbs within a condition so that each mouse acted as its own control. This method was used in three ways. First, rather than pooling left and right hindlimb data and comparing these data between conditions, we separated left and right gait metrics and compared these datasets within the same condition. Briefly, as a first step in this method, stride-to-stride gait metrics were plotted as a function of stride velocity. Selection of appropriate regression model was achieved as outlined in detail before^24^. Using a sum of squares F test, the fit for models from simple to more complex were compared using linear, one phase association and two phase association models for each metric. This was done for different ranges of speed within each model and for different cohorts of mice. Speed ranges were tested by omitting increasing ranges of slowest velocities (starting at a bin of 0–1 cm/s and increasing by 1 cm/s) and/or the highest (starting at 25 cm/s and decreasing by 1 cm/s). The simplest regression model that consistently captures each gait metric across cohorts was then selected (see Supplementary Table 2 in^24^). The speed range for which these models consistently worked across groups was 3–16 cm/s. The simplest model was chosen in order to avoid overfitting the data^52^ as that increases the risk of detecting statistically significant but biologically irrelevant differences. As described previously, we used the run’s test to determine whether curves fit by nonlinear regression would deviate systematically from the data. As outlined by Broom *et al*.^24^, this relatively simple approach is valid when mice are used as their own control (mature age and higher) or when groups are very carefully matched. More complex models may be necessary when these requirements are not met. We used a p value for the F-test of <0.001^24^. A stringent p value is used to ensure analyses detect differences that are biologically meaningful, accounting for potential over-fitting of datasets and pseudo-replication^24^. For the linear models the F test captured both slope and Y intercept. Of note, we are primarily interested in a speed range that is most relevant for human walking, as related to patient outcomes and daily function. The intent of the study was to use the species specific and cohort specific datasets that we obtained through direct measurements and analyze data within species. On the runway used in our studies, gait in the 3–16 cm/s range represents walking as well as trotting as verified with video analysis. Temporal and spatial coupling of the hindlimbs at baseline conditions was alternating for the datasets in mice as we show later, and as it is during normal walking in humans. The dynamics of the gait curves (see for example stance curves) in mice at this 3–16 cm/s speed range strongly resemble those in humans instructed to walk (this study and Broom *et al*.^24^). We used the same parameters for the current study, except for the analysis of stance time. If the stance curve would not fit, automatic outlier elimination was omitted to accommodate the smaller number of data points in the separate left and right datasets. Also, the speed range for the MPTP baseline forelimb dataset was limited to 4–16 cm/s as there were too few data points present at speeds slower than 4 cm/s. Logarithmic transformation of the stance time data and application of a linear model provided consistent results without any adjustments.

The second modification included the incorporation of step length, which is defined as the distance at which one limb is placed in front of the opposing limb. Step length, unlike stride length, accounts for relative placement of left and right limbs (Fig. 4a). We analyzed step length as a function of stride velocity using the same analysis parameters as for stride length^24^.

The third modification related to forelimb data. Given known asymmetries in the forelimb paw placement test in unilateral 6-OHDA rat models, we applied the analyses used for the hindlimbs to forelimb metrics (stride length, swing time, stance time, and step length).

This approach is valid when comparing data from the same mice (starting at the mature age and up) or from very carefully matched mature cohorts. Under these conditions, within group variation is small (Broom, *et al*. page 7, paragraph 2, Fig. 2c–g). We performed a series of additional analyses to further validate this approach for the current datasets and methodology. To illustrate that the datasets used in this study are homogeneous, we randomly divided mice in the 6-OHDA cohort into two equal sized groups (group A and group B, N = 9 each). We do not expect to see differences between datasets of group A and group B in either the pre- or post-lesion conditions. We compared left leg step length datasets for groups A and B. No significant difference was found between regression curves of the two groups (Supplemental Fig. 2S).

To test whether data of a single mouse was independent, i.e. randomly distributed when ordered by stride sequence (null hypothesis: there is no pattern to the data sequence), we performed a runs test (6-OHDA cohort; pre-lesion; step length). Using the mean as a reference value, the runs test failed to reject the null hypothesis (Z = −1.18, p = 0.23; N = 1, n = 18). We also graphed left and right step length data from one mouse from the 6-OHDA cohort both pre and post lesion, in order to visualize the typical distribution of data points and confirm that they do not cluster (Supplemental Fig. 3S).

To further verify that the results of the above tests in the 6-OHDA model were not driven by multiple data points of a few mice (pseudo-replication), we analyzed average left and right step length, stride length, swing time and stance duration in each mouse as a function of average speed using the same approach as outlined above (F-test). In addition, we analyzed differences in left and right average step length, stride length, swing time and stance duration using a two-tailed, paired-t test. As outlined in detail in Broom *et al*.^24^, working with averaged datasets when studying gait is feasible only when the range in stride to stride speed does not vary between groups. This is often not the case when studying disease models. Even when speed does not change, averaged datasets are hard to interpret as can be observed when comparing stance data in Fig. 1 g ii, with Supplemental Fig. 1Sd*iii*.

Analysis 2: Quantifying asymmetries between conditions: We next compared the step length datasets of each leg prior to and after induction of parkinsonism using the same approach as outlined above. This was done to confirm the side with the shorter step length.

Analysis 3: Quantifying asymmetries within and between conditions: The above analyses either compare data between paired limbs within a single condition or data from one limb between conditions, but not between limbs AND between conditions. To address this limitation, we used polar plots to visualize differences in spatial or temporal metrics of sets of hind- or forelimbs between conditions, and applied circular statistics to measure these differences. This approach is customary for analyzing timing in rhythmic behaviors^25–27,38^ but is not commonly used for spatial parameters. For spatial analyses, we calculated the ratio of step length to opposing stride length (Fig. 4b). We referred to these measures as spatial alternation ratios. For the temporal analyses, we calculated the ratio between the time offset of sequentially opposing footfalls and cycle duration of pairs of fore- or hindlimbs (Fig. 4c). We referred to these measures as temporal alternation ratios. Spatial and temporal ratios were then treated as circular datasets, with 0 and 1 representing identical events (placement at the same horizontal location as the opposite step, or placement at the same time as the opposite step). Data were plotted on the circular axis of polar plots, with average values represented by angle of radial lines, the lengths of which represent the r value corresponding to strength of clustering. These analyses were performed in MATLAB using functions from the toolbox “CircStat for Matlab”^53^. Data were not visualized in a speed dependent manner. However, two analyses were performed. First, data within the relevant range of 3–16 cm/s were analyzed. For a second analysis, data were divided into two speed bins, 3–10 and 10–16 cm/s and analyzed separately. The Watson-Williams test was used to quantify differences in datasets between conditions. This test measures the equality of means of two or more samples where numbers represent angles in radians. For these analyses the significance level was set at 0.05.

#### Histological processing and analysis

Following behavioral testing, mice were transcardially perfused with phosphate buffered saline (PBS) followed by 10% formalin under chloral hydrate anesthesia (500 mg/kg i.p.). Dissection, processing, immunohistochemistry for tyrosine hydroxylase (TH sheep, AB152, 1:5000; Lot 2668078; Millipore) and image analysis to measure TH immunoreactivity in the striatum, a measure for the severity of nigral dopaminergic cell loss, were performed per standard protocol^24^. Subcutaneous MPTP (or 6-OHDA where injection involves the ventral tegmental area) can affect mesolimbic as well as nigrostriatal dopamine systems^54,55^. In addition, TH immunoreactivity in the striatum does not necessarily provide a measure of the depletion of dopamine. Despite these limitations, we chose to focus only on the measure of characteristic loss of TH immunoreactivity in the striatum. This was done strictly as a screening measure in order to ensure uniformity of the groups used for gait analysis as outlined above^24^ and was not intended to represent a complete picture of the resulting injury of either toxin. A 80% or greater decrease in TH-IR density in the ipsilateral striatum compared to the contralateral side was required for the 6-OHDA datasets (18 out of 32 mice; average loss 94 +/− 6%) and a 65% or greater decrease in TH-IR density in the striatum compared to control animals (17 out of 19; average loss 77 +/− 10%) was required in the MPTP group.

### Human datasets

#### Subjects

To determine whether the approach described above could also be applied to detect asymmetries in gait metrics in humans, we analyzed spatial and temporal gait metrics of overground walking in healthy subjects and subjects with Parkinson’s disease (clinical trials.gov: NCT02994719). This study was approved by the Committee on Clinical Investigations, the institutional review board for research involving human subjects at Beth Israel Deaconess Medical Center. All methods in this study involving human subjects were carried out in accordance with relevant regulations and guidelines. Subjects provided written informed consent prior to enrollment. Here we only report results from male subjects, as recruitment of female subjects with PD was too low for a separate analysis, which is necessary given differences in spatial gait metrics between males and females^24^.

Subjects included 29 patients with PD (age range from 55 to 77 years) and 13 age and sex matched healthy control subjects (age range from 47 to 77 years). All subjects were examined by a neurologist to confirm a clinical diagnosis of PD (UK Parkinson’s disease society brain bank criteria^56^ or, for the control subjects, to confirm absence of parkinsonism or other neurological abnormalities. Subjects with PD were tested during the ON medication state and we calculated levodopa equivalent dose^57^. Two subjects who had undergone bilateral implantation of deep brain stimulation electrodes in the subthalamic nucleus two years prior to enrollment were tested with the stimulator turned on at the best therapy settings. Nine subjects with PD reported a history of dyskinesias on the MDS-UPDRS part IV. None of these subjects had dyskinesias during gait testing. One subject with PD, who did not report a history of dyskinesias was observed through video analysis to have dyskinesias during gait testing. Data was analyzed and displayed with this subject included. A separate analysis with this subject excluded did not change the outcomes.

#### Gait assessment and analysis

In order to obtain gait metrics covering a wide range of walking speeds, subjects were instructed to walk across a 16 foot gait mat (Zeno, Protokinetics, Havertown, PA, USA; 16 × 2 feet; 576 pressure sensors per square foot; spatial resolution 0.5 by 0.5 inches; temporal resolution 1/120 second) for a total of 6 trials which included preferred speed (2 trials) and 1 trial each of slow, very slow, fast and very fast speeds^24^. Stride velocity, step length, stride length, swing time, and stance time were captured from pressure sensors in the mat using PKMAS software^24^.

#### Statistical analyses

Asymmetries in subjects with PD are expected to occur with almost equal frequency on either side, with the side of presentation weakly related to handedness^58,59^. This in contrasted with the 6-OHDA mouse model in which parkinsonism is induced on the same side in all mice. Therefore, in PD patients, asymmetries between the left versus right datasets are likely to be obscured at the group level. To circumvent this problem, a rater who was blinded to diagnosis and clinical details first assigned subjects as “asymmetric” or “symmetric” based upon visual assessment of video recordings from walking trials on the gait mat. In subjects labeled as “asymmetric”, the rater then visually determined the side with the shorter or longer step lengths. No clinical asymmetries were observed in the control group, whereas clinically visible asymmetries were noted in 9 out of 29 PD patients. In the control group and in PD subjects without obvious gait asymmetry, all gait parameters were organized into true left or right sides. In PD subjects with asymmetries, 5 subjects presented with shorter steps on the right and 4 presented with shorter steps on the left. In the latter 4 subjects, we then re-assigned all left metrics to the right and all right metrics to the left for the purpose of group analyses.

To examine asymmetries in gait metrics we then analyzed the datasets in 3 different ways:

Analysis 1: In the first analysis, we tested the hypothesis that temporal or spatial asymmetries are present in the PD group and not in the control group. Similar to Analysis 1 in the mouse models, within each group we depicted each of the gait metrics as a function of gait velocity for the (assigned) left and right sides. We then determined the simplest model that best fit the data. Data points between 0.3 m/s and 1.5 m/s were analyzed as insufficient data points, representing only a few subjects, were present outside this range. We used a linear model for step and stride length, for swing time, and a one phase association model for stance time. Stance time data was also transformed logarithmically (Y = 1-log(stance time), X = (log speed) +1) and fit to a linear model. No constraints were applied to these models. Next, we applied an F test to assess whether regression curves differed between longer/shorter or left/right sides for each parameter. This simple analysis was feasible given that the left and right datasets used for comparison were derived from the same individuals. Direct comparisons of datasets between groups would require more complex approaches, which is beyond the goals of the current study. Similar to analyses in mice, significance level was set conservatively at 0.001 to avoid statistically significant but biologically insignificant results and to accommodate for multiple data points within each subject.

Analysis 2: We next assessed whether asymmetries in gait metrics in the PD cohort were manifestations of Parkinson’s disease subtype. The rationale for this analysis is that only a subgroup of the PD cohort displayed obvious asymmetries, and that swing time asymmetries have been attributed to PIGD subtype^6^. We divided the PD cohort into groups with tremor dominant (TD) symptoms, with postural instability and gait difficulty (PIGD) or with mixed phenotype based on subjects’ scores on the MDS-UPDRS as outlined by Stebbins *et al*., 2013^28^ in the ON-medication state. We then assessed whether the left and right spatial and temporal datasets differed within the TD and PIGD groups, using the same analyses as above.

Analysis 3 For a third set of analyses, we divided the PD cohort into a group with and a group without clinical gait asymmetry. We then used a similar approach as in the first analysis to detect which asymmetries in gait metrics correspond to the clinical video assessment.

Summary statistics: In addition, to assess for group differences in age, MDS-UPDRS, height, weight or cognitive performance (MoCA), we used a 2 tailed unpaired T test with Welch’s correction to account for unequal sample sizes. Weight was not available for 1 subject in the PD group and 1 subject in the control group. Height was not available for 1 subject with PD. Significance level was set at 0.05 and no corrections were made for multiple comparisons.

Finally, for each PD subject asymmetry ratios were calculated for rigidity, bradykinesia, swing time and step length. Rigidity and bradykinesia measures of the left and right side were taken from the sub scores of the MDS-UPDRS, whereas an average was calculated for the gait metrics from all available left or right data points. We then used the Pearson correlation (two tailed, p 0.05) to assess whether asymmetries in rigidity or bradykinesia correlated with asymmetries in swing time or step time.

### Code availability

The custom MATLAB code used to score mouse data that was collected on a custom runway is available from the corresponding author upon reasonable request.

## Supplementary information

Supplementary Information

## Data Availability

Supporting data for findings reported in this study are available from the corresponding author upon reasonable request.
