# Supplementary material for: Translational methods to detect asymmetries in temporal and spatial walking metrics in parkinsonian mouse models and human subjects with Parkinson’s disease
Source: Sci Rep. 2019 Feb 21;9:2437. doi: 10.1038/s41598-019-38623-6 (PMC6385183; doi:10.1038/s41598-019-38623-6)
Supplement: Supplementary file 1 — Supplementary Information [file 41598_2019_38623_MOESM1_ESM.pdf]

Translational methods to detect asymmetries in temporal and spatial walking metrics in parkinsonian mouse models and human subjects with Parkinson's disease

Lauren Broom, Audrey Worley, Fay Gao, Laura D. Hernandez, Christine E. Ashton, Ludy C. Shih, Veronique G. VanderHorst*

Department of Neurology, Division of Movement Disorders, Beth Israel Deaconess Medical Center and Harvard Medical School, 3 Blackfan Circle, Boston, MA 02115, USA.

Corresponding author:

*Veronique VanderHorst MD PhD

Beth Israel Deaconess Medical Center

Center for Life Sciences, CLS 706

3 Blackfan Circle

Boston MA 02115

Phone: 617 735 3201

Figure 1S

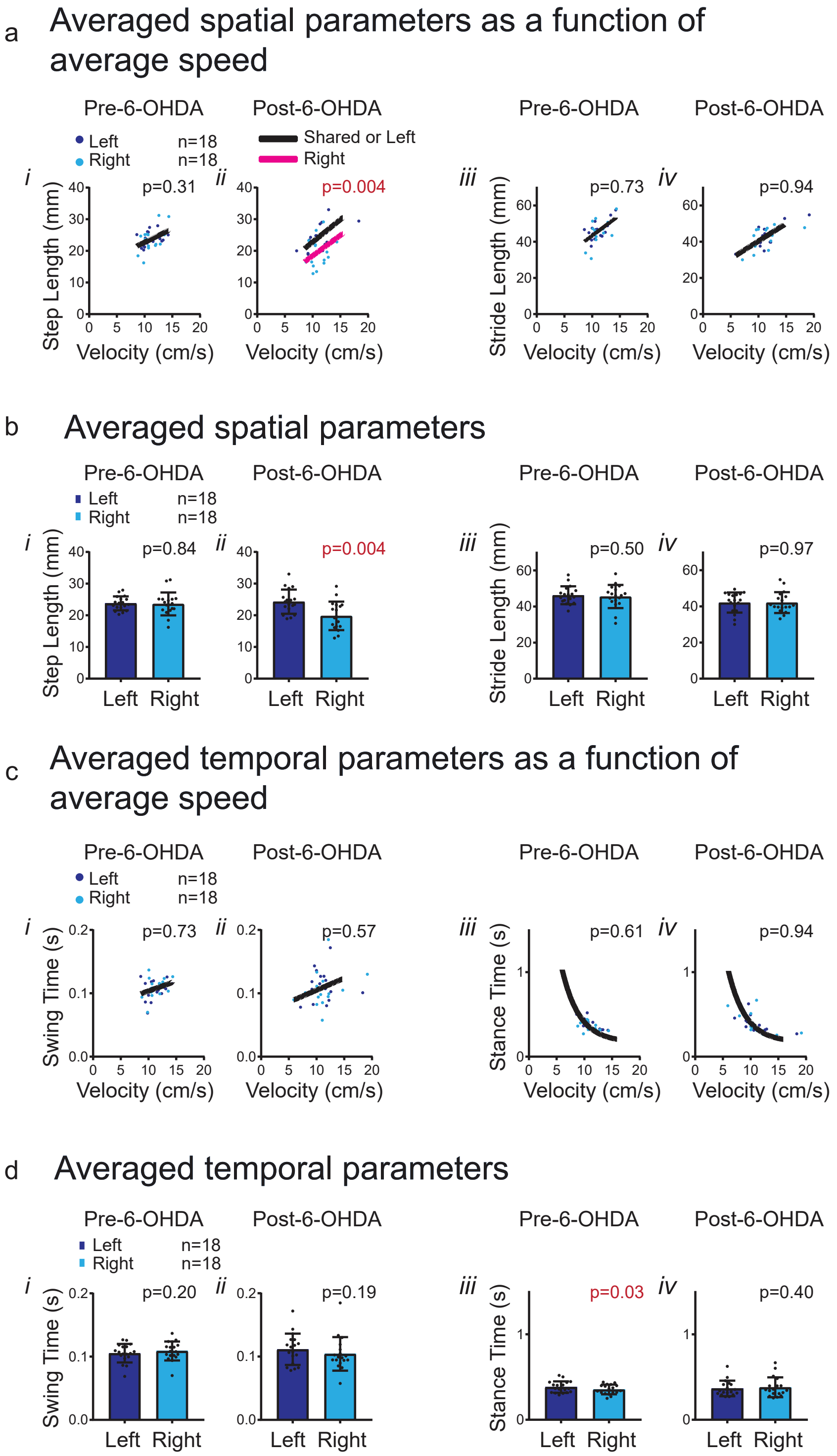

Figure 1S: Alternative analyses of gait symmetry in a unilateral 6-OHDA model Averaged spatial metrics step length (a i ii) and stride length (a iii iv) and temporal metrics swing time (c i ii) and stance time (c iii iv) of the left (dark blue dots) and right (cyan dots) hindlimbs as a function of averaged velocity following induction of parkinsonism. In a and c, a combination of black and magenta lines represent a significant difference in datasets. A single black line represents the single fit of pooled datasets when they are not significantly different (F-test, p value set at 0.001; Supplemental Table 2S). Averaged spatial metrics step length (b i ii) and stride length (b iii iv) and temporal metrics swing time (d i ii) and stance time (d iii iv) of the left (dark blue bars) and right (cyan bars) hindlimbs following induction of parkinsonism. Data was compared using a paired t-test (2-tailed) with p value set at 0.05; Supplemental Table 3S).

Figure 2S

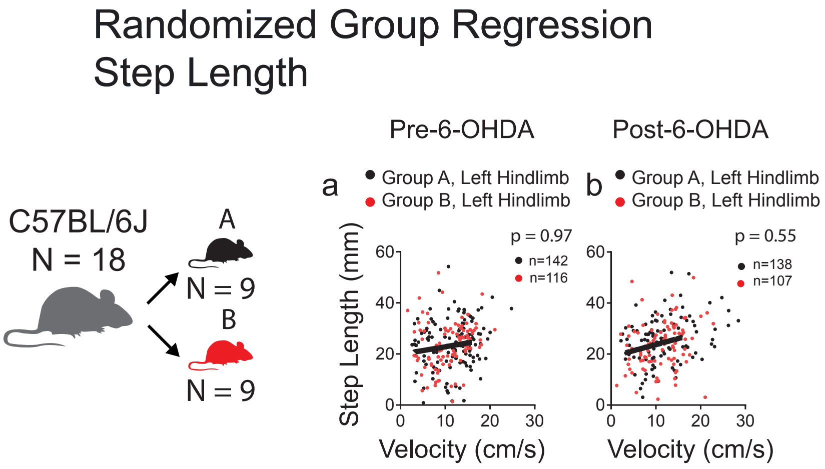

Figure 2S: Randomized group regression

Left hind-limb step length metrics in pre-lesion (a) and post lesion (b) conditions for the 6-OHDA cohort randomly divided into two groups. Step length metrics as a function of velocity represented for the two randomized groups as with group A (black dots) compared to group B (red dots).

Figure 3S

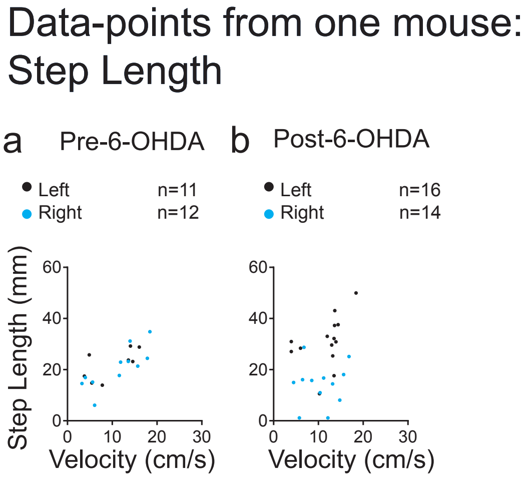

Figure 3S: Distribution of step length data from one mouse Left and right hind-limb step length metrics of one mouse from the 6-OHDA cohort in pre-lesion (a) and post lesion (b) conditions. Step length metrics as a function of velocity represented for a randomly selected mouse with left (black dots) and right (blue dots) step length.

Figure 4S

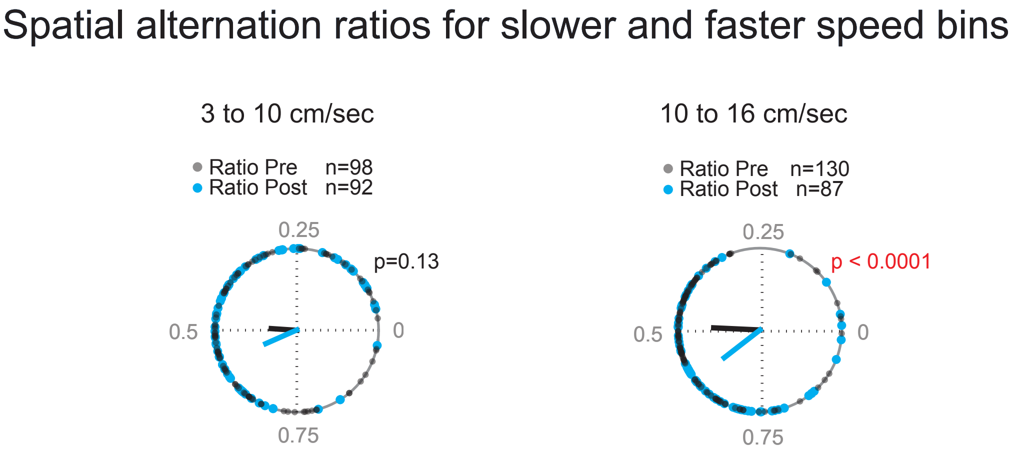

Figure 4S: Spatial alternation ratios at upper and lower speed ranges Spatial alternation ratio of hindlimbs plotted on the circular axis of polar plots (as in Figure 2). Data is divided into two speed bins (3-10 or 10-16cm/s). The Watson-Williams test was used to determine differences in group means (p value set at 0.05; Supplemental Table 6S).

Supplemental Table 1S

Comparison between gait metrics of left and right hind- or forelimbs at baseline and in experimental PD or control conditions

		**Baseline**				**Experimental**			
		**Hindlimbs**		**Forelimbs**		**Hindlimbs**		**Forelimbs**	
**Model**		F (DFn, DFd)***	p	F (DFn, DFd)	p	F (DFn, DFd)	p	F (DFn, DFd)	p
**6-OHDA**	Step length	0.01 (2, 384)	0.99	1.0 (2, 366)	0.36	18.86 (2, 333)	<0.0001	16.27 (2, 301)	<0.0001
	Stride length	1.00 (2, 446)	0.37	0.49 (2, 422)	0.61	1.40 (2, 375)	0.25	6.98 (2, 348)	0.0011
	Swing time	1.26 (2, 446)	0.28	0.59 (2, 422)	0.55	1.21 (2, 375)	0.30	2.13 (2, 342)	0.12
	Stance time	1.73 (4, 442)	0.14	0.23 (4, 425)*	0.92	0.41 (4, 356)	0.80	3.00 (4, 311)	0.02
	Log stance time	4.40 (2, 525)	0.01	1.75 (2, 532)	0.18	0.53 (2, 485)	0.59	6.463 (2, 435)	0.0017
		F (DFn, DFd)	p	F (DFn, DFd)	p	F (DFn, DFd)	p	F (DFn, DFd)	p
**MPTP**	Step length	2.36 (2, 344)	0.10	1.65 (2, 324)	0.19	0.05 (2, 418)	0.95	0.64 (2, 410)	0.53
	Stride length	1.72 (2, 383)	0.18	0.16 (2, 364)	0.85	0.44 (2, 447)	0.64	0.89 (2, 431)	0.41
	Swing time	3.56 (2, 383)	0.03	0.96 (2, 364)	0.38	2.42 (2, 447)	0.09	1.7 (2, 423)	0.18
	Stance time	1.61 (4, 396)*	0.17	0.21 (4, 310)**	0.93	1.50 (4, 461)	0.20	0.15 (4, 415)	0.96
	Log stance time	5.4 (2, 585)	0.005	0.49 (2, 592)	0.62	0.48 (2, 628)	0.62	1.426 (2, 646)	0.24
		F (DFn, DFd)	p	F (DFn, DFd)	p	F (DFn, DFd)	p	F (DFn, DFd)	p
**Control**	Step length	0.76 (2, 307)	0.47	1.34 (2, 321)	0.26	0.9 (2, 317)	0.41	3.33 (2, 301)	0.04
	Stride length	0.03 (2, 354)	0.97	0.12 (2, 372)	0.88	0.02 (2, 361)	0.98	0.12 (2, 341)	0.89
	Swing time	0.09 (2, 354)	0.92	0.12 (2, 372)	0.89	0.1 (2, 361)	0.91	0.03 (2, 333)	0.97
	Stance time	0.35 (4, 350)	0.85	0.58 (4, 382)*	0.67	0.45 (4, 357)*	0.77	2.22 (4, 360)	0.07
	Log stance time	0.13 (2, 442)	0.88	0.05 (2, 462)	0.95	4.00 (2, 593)	0.02	0.55 (2, 503)	0.58

* No outlier detection applied.

* *Speed range from 4-16cm/s due to insufficient data points <3cm/s.

* * *F test result (Dfn = number of parameters fit by the regression, DFd = data points minus number of parameters fit by regression)

A p level of < 0.001 is considered significant.

Supplemental Table 2S
Comparison between average left and average right hindlimb metrics
as a function of average stride velocity at baseline and in experimental (6-OHDA) condition

	**Baseline**		**Experimental**	
	F (DFn, DFd)	p	F (DFn, DFd)	p
Step length	1.22 (2, 32)	0.31	6.71 (2, 32)	0.004
Stride length	0.32 (2, 32)	0.73	0.007 (2, 30)	0.94
Swing time	0.31 (2, 32)	0.73	0.57 (2, 30)	0.57
Stance time	0.50 (2, 32)	0.61	0.006 (2, 26)	0.94

Supplemental Table 3S
Comparison between average left and average right hindlimb metrics at baseline and in experimental (6-OHDA) condition

	**Baseline**							**Experimental**						
		**Left**		**Right**		**Average metrics**			**Left**		**Right**		**Averaged metrics**	
	N	Mean	SD	Mean	SD	paired t (2-tailed)	p	N	Mean	SD	Mean	SD	paired t (2-tailed)	p
Step length	18	23.77	2.20	23.61	3.62	t=0.20 df=17	0.84	18	24.3	3.82	19.82	4.50	t=3.35 df=17	0.004
Stride length	18	46.26	4.96	45.52	6.44	t=0.68 df=17	0.50	18	42.1	5.57	42.07	5.79	t=0.03 df=17	0.97
Swing time	18	0.11	0.01	0.11	0.02	t=1.3 df=17	0.20	18	0.11	0.02	0.10	0.03	t=1.38 df=17	0.19
Stance time	18	0.38	0.07	0.35	0.06	t=2.38 df=17	0.03	18	0.37	0.09	0.38	0.12	t=0.87 df=17	0.40

Supplemental Table 4S

Comparison of step length of each limb between baseline and experimental PD or control conditions

		Hindlimbs				Forelimbs			
		Left		Right		Left		Right	
**Model**		F (DFn, DFd)	p	F (DFn, DFd)	p	F (DFn, DFd)	p	F (DFn, DFd)	p
**6-OHDA**	Step length	0.65 (2, 361)	0.52	12.15 (2, 356)	<0.0001	3.86 (2, 330)	0.02	10.49 (2, 337)	<0.0001
**MPTP**	Step length	3.40 (2, 378)	0.03	5.81 (2, 384)	0.003	8.76 (2, 375)	0.0002	0.67 (2, 359)	0.51
**Control**	Step length	4.97 (2, 317)	0.0075	3.03 (2, 307)	0.05	0.69 (2, 312)	0.50	3.2 (2, 310)	0.04

A p level of < 0.001 is considered significant.

Supplemental Table 5S

Comparison of spatial and temporal coupling of hind- or forelimbs between baseline and experimental PD or control conditions

**Spatial coupling**	**Hindlimbs**							**Forelimbs**						
	Baseline			Experimental			Watson-Williams	Baseline			Experimental			Watson-Williams
**Model**	Mean	SD	r	Mean	SD	r	p	Mean	SD	r	Mean	SD	r	p
**6-OHDA**	0.50	0.18	0.39	0.61	0.18	0.33	<0.0001	0.51	0.11	0.64	0.59	0.11	0.60	<0.0001
**MPTP**	0.50	0.17	0.41	0.49	0.17	0.41	0.46	0.51	0.13	0.65	0.49	0.14	0.59	0.27
**Control**	0.50	0.16	0.52	0.48	0.17	0.42	0.20	0.51	0.14	0.63	0.52	0.15	0.56	0.61

**Temporal coupling**	**Hindlimbs**							**Forelimbs**						
	Baseline			Experimental			Watson-Williams	Baseline			Experimental			Watson-Williams
**Model**	Mean	SD	r	Mean	SD	r	p	Mean	SD	r	Mean	SD	r	p
**6-OHDA**	0.50	0.13	0.66	0.49	0.16	0.52	0.54	0.51	0.14	0.78	0.59	0.14	0.75	0.18
**MPTP**	0.52	0.13	0.65	0.50	0.13	0.67	0.27	0.50	0.11	0.77	0.51	0.12	0.72	0.26
**Control**	0.51	0.11	0.74	0.51	0.13	0.65	0.91	0.52	0.12	0.70	0.50	0.11	0.77	0.17

* Mean and SD refer to circular mean and circular SD.

A p level of < 0.05 is considered significant.

Supplemental Table 6S

Spatial and temporal couping of hindlimbs at slower and faster walking speeds

**Spatial**		**Baseline**			**Experimental**			**Watson-Williams**
	**Speed Bin**	Mean	SD	r	Mean	SD	r	p
**6-OHDA**	**3 to 10 cm/second**	0.49	0.19	0.31	0.43	0.17	0.41	0.13
	**10 to 16 cm/second**	0.51	0.17	0.45	0.43	0.17	0.45	0.00001
**MPTP**	**3 to 10 cm/second**	0.45	0.19	0.25	0.39	0.20	0.25	0.21
	**10 to 16 cm/second**	0.52	0.15	0.57	0.51	0.14	0.59	0.82
**Control**	**3 to 10 cm/second**	0.54	0.18	0.39	0.56	0.20	0.23	0.63
	**10 to 16 cm/second**	0.51	0.15	0.57	0.51	0.15	0.58	0.92

**Temporal**		**Baseline**			**Experimental**			**Watson-Williams**
	**Speed Bin**	Mean	SD	r	Mean	SD	r	p
**6-OHDA**	**3 to 10 cm/second**	0.49	0.20	0.24	0.52	0.19	0.31	0.55
	**10 to 16 cm/second**	0.51	0.08	0.87	0.49	0.11	0.76	0.24
**MPTP**	**3 to 10 cm/second**	0.49	0.19	0.32	0.49	0.17	0.46	0.88
	**10 to 16 cm/second**	0.52	0.08	0.87	0.50	0.07	0.89	0.24
**Control**	**3 to 10 cm/second**	0.51	0.15	0.54	0.52	0.18	0.39	0.88
	**10 to 16 cm/second**	0.51	0.07	0.91	0.50	0.07	0.91	0.48

Supplemental Table 7S

Comparison of left and right gait metrics in subjects with Parkinson's disease or control subjects

**Analysis 1**	**Parkinson's Disease**		**Healthy Control**	
Number of subjects	29		13	
	F (DFn, DFd)	p	F (DFn, DFd)	p
Step length (m)	12.92 (2, 1603)	<0.0001	1.12 (3, 547)	0.34
Stride length (m)	0.02 (2, 1575)	0.98	0.27 (2, 587)	0.76
Swing time (s)	0.98 (2, 1603)	0.38	3.20 (2, 549)	0.04
Stance time (s)	2.57 (3, 1558)	0.05	0.34 (3, 586)	0.79
log Stance time (s)	0.48 (2, 1811)	0.62	0.21 (2, 745)	0.81

**Analysis 2**	**PD: Postural Instability Gait Disorder**		**PD: Tremor Dominant**	
Number of subjects	12		15	
	F (DFn, DFd)	p	F (DFn, DFd)	p
Step length (m)	7.86 (2, 716)	0.0004	9.16 (2, 770)	0.0001
Stride length (m)	0.04 (2, 716)	0.96	0.53 (2, 658)	0.59
Swing time (s)	12.54 (2, 716)	<0.0001	0.17 (2, 770)	0.85
Stance time (s)	2.90 (3, 705)	0.03	1.13 (3, 722)	0.34
log Stance time (s)	3.99 (2, 760)	0.02	0.17 (2, 925)	0.84

**Analysis 3**	**PD: Clinical Asymmetry**		**PD: No Clinical Asymmetry**	
Number of subjects	9		20	
	F (DFn, DFd)	p	F (DFn, DFd)	p
Step length (m)	44.17 (2, 494)	<0.0001	4.51 (2, 1123)	0.01
Stride length (m)	1.12 (2, 472)	0.33	0.50 (2, 1099)	0.60
Swing time (s)	0.03 (2, 496)	0.97	0.29 (2, 1102)	0.75
Stance time (s)	1.60 (3, 497)	0.18	0.32 (3, 1034)	0.81
log Stance time (s)	0.32 (2, 552)	0.73	0.47 (2, 1255)	0.62

A p level of < 0.001 is considered significant.

Supplemental Table 8S

Correlation of Asymmetry ratios of clinical and gait measures in subjects with Parkinson's disease

N=29 subjects with Parkinson's disease	r	95% confidence interval	R squared	Pearson correlation P (two-tailed)
Bradykinesia vs Swing time	-0.06	-0.4156 to 0.3153	0.0034	0.77
Bradykinesia vs Step length	-0.03	-0.3907 to 0.3418	0.0008	0.88
Rigidity vs Swing time	-0.05	-0.4073 to 0.3243	0.0023	0.81
Rigidity vs Step length	-0.08	-0.4329 to 0.2962	0.0062	0.68
Swing time vs Step length	-0.23	-0.5533 to 0.1446	0.0549	0.22

A p level of < 0.05 is considered significant.
